# Relationship between follicular size and developmental capacity of oocytes under controlled ovarian hyperstimulation in assisted reproductive technologies

**DOI:** 10.1002/rmb2.12382

**Published:** 2021-03-23

**Authors:** Isao Tamura, Mai Kawamoto‐Jozaki, Taishi Fujimura, Yumiko Doi‐Tanaka, Haruka Takagi, Yuichiro Shirafuta, Yumiko Mihara, Toshiaki Taketani, Hiroshi Tamura, Norihiro Sugino

**Affiliations:** ^1^ Department of Obstetrics and Gynecology Yamaguchi University Graduate School of Medicine Ube Japan

**Keywords:** blastocyst, follicular size, ICSI, IVF, oocyte development

## Abstract

**Purpose:**

We investigate the relationships between oocyte developmental capacity and follicular size of its origin in Japanese women: those undergoing conventional IVF (cIVF) and ICSI, respectively.

**Methods:**

A total of 3377 follicles were punctured separately and were classified into three groups (large, medium, and small) by their diameters. A total of 1482 retrieved oocytes were individually cultured and received cIVF or ICSI. The oocytes receiving ICSI were denuded and the number of mature (MII) oocytes was counted.

**Results:**

The oocyte retrieval rates and the proportion of MII oocytes were significantly lower in small follicles than in large follicles. Under cIVF, the fertilization rate was significantly lower in oocytes from small follicles than large follicles. Under ICSI, the fertilization rate for MII oocytes was not significantly related to follicular size. Follicular size was not significantly related to the development potential to blastocyst and pregnancy rate for either the cIVF oocytes or the ICSI oocytes.

**Conclusions:**

Although the fertilization rate by cIVF is low in oocytes from small follicles due to the lower proportion of mature oocytes, their development potential is comparable to that of oocytes from larger follicles if they could be fertilized. Under ICSI using mature oocytes, their development potential is not related to follicular size.

## 1 INTRODUCTION

Assisted reproductive technologies (ARTs) have been widely used to treat infertility for the past 30 years. The number of ART cycles has dramatically increased each year.^1^ Controlled ovarian hyperstimulation (COH) with exogenous gonadotropins has been frequently used in vitro fertilization (IVF) programs. The administration of exogenous gonadotropins maintains high levels of follicle‐stimulating hormone (FSH), which suppresses follicular atresia and induces multiple follicular growth. This allows the retrieval of multiple oocytes and thereby multiple embryos in a single IVF cycle.^2^ However, not all follicles develop in synchrony, and there are always different sizes of follicles.^3^ It is well known that oocyte maturation progresses during follicular development under FSH stimulation.^4^ Therefore, there is a possibility that the developmental capacity of oocytes retrieved during COH depends on the size of the follicles from which they came. In fact, previous reports showed that the oocyte retrieval rate was lower for small follicles than it was for large follicles.^5^, ^6^ Some reports have shown that oocytes from small follicles have a reduced rate of fertilization or embryo development.^6^, ^7^, ^8^, ^9^ However, other studies did not find such differences.^5^, ^10^, ^11^ Therefore, the relationship between follicular size and developmental capacity of oocytes is still controversial. These discordant findings might be due to differences in patient characteristics, methods of oocyte insemination (conventional IVF [cIVF] or intracytoplasmic sperm injection [ICSI]), and the assessment of developmental capacity. In most previous studies, the developmental capacity of oocytes has been evaluated by assessing the cleavage rate or the quality of cleavage‐stage embryos, but not blastocysts. However, blastocyst transfer is becoming more common because it results in a higher implantation rate than does the transfer of cleavage‐stage embryos.^12^ Because blastocyst quality is strongly associated with the pregnancy rate,^13^ the developmental capacity of oocytes should be determined by assessing the quality or formation rate of blastocysts. Recently, Wirleitner et al reported that when oocytes are fertilized by ICSI, those from small follicles have the same developmental capacity up to the blastocyst stage as those from large follicles.^14^ However, none of previous studies investigated the correlation between follicular size and the developmental capacity up to the blastocyst stage under cIVF.

Furthermore, because the efficiency of ART is different between races,^15^, ^16^ it remains unclear whether these findings also apply to Japanese patients. According to the latest report from the International Committee Monitoring Assisted Reproductive Technologies (ICMART), Japan is the largest user of ART worldwide in terms of annual number of treatment cycles performed.^1^, ^17^, ^18^ Therefore, it is important to conduct a study to clarify the relationship between follicular size and the development capacity of oocytes in Japanese women. In this study, we individually cultured the retrieved oocytes and investigated the size/development relationship by examining blastocyst development and pregnancy rate of oocytes from groups of Japanese women undergoing cIVF or ICSI.

## 2 MATERIAL AND METHODS

### 2.1 Study population

This retrospective cohort study included 176 patients with infertility who underwent oocytes retrieval for cIVF or ICSI at Yamaguchi University Hospital between June 2014 and June 2020. Informed consent was obtained from all the patients in this study. The study design was reviewed and approved by the institutional review board of Yamaguchi University Hospital.

### 2.2 Controlled ovarian hyperstimulation protocol and oocyte retrieval

Controlled ovarian hyperstimulation was performed using standard gonadotropin releasing hormone agonist (GnRHa) / FSH protocols. Nasal spray GnRHa (900 μg/d) was given from the mid‐luteal phase in the previous cycle to continuously suppress pituitary gonadotropin secretion until the injection of HCG (10 000 IU). COH was initiated from the 2nd day of the IVF‐ET cycle by injection of 225 IU FSH for 3 days, followed by a daily injection of 150 IU HMG. When more than three leading follicles reached 18 mm or more, HCG was injected for ovulation induction. Oocyte retrieval was carried out 35 hours after HCG injection. For patients who showed poor response to COH in the previous IVF cycle, they underwent short GnRH agonist protocol. In this protocol, COH and GnRHa were simultaneously initiated from the 2nd day of the IVF‐ET cycle. 300 IU FSH or 300 IU HMG was daily injected until the day of HCG injection.

Each follicular size was measured and recorded before the oocyte aspiration. Follicles were classified into three groups according to their diameters as measured by transvaginal ultrasonography: large follicle (≧18 mm), medium follicle (13‐17 mm), and small follicle (≦12). Each follicle was aspirated separately, and the retrieved oocyte was individually cultured to correlate their developmental outcomes with follicular size. For the oocytes receiving ICSI, oocytes were denudated, and the number of metaphase II (MII) oocytes was counted. Only MII oocytes were proceeded to sperm injection.

### 2.3 Fertilization and embryo transfer

The semen samples were collected by masturbation, and the motile sperm was collected by swim‐up technique as reported previously.^19^ Fertilization was performed using either standard insemination (cIVF) or ICSI with Piezo‐assisted ICSI system (Prime Tech Ltd). In the early years of this study, all oocytes were subjected to cIVF. From 2017 onwards, ICSI was introduced to patients with severe male factor or fertilization failure. If the sperm concentration is low (motile sperm concentration is <5 × 10^5^/mL after swim‐up) or three was a history of fertilization failure (previous fertilization rate under cIVF was <25%), all retrieved oocytes underwent ICSI procedure. If the previous fertilization rate under cIVF was between 26% and 50%, half of the retrieved oocytes were allocated to receive ICSI, and the other half were allocated to receive cIVF. This was designated as “split” as reported previously.^20^ Fertilization was confirmed on day 1 (17‐19 hours after insemination) with the presence of two pronuclei. The cleavage of embryo was assessed on day 2. The blastocyst formation and its quality were assessed on day 5 according to the established score guidelines.^21^ A high‐quality blastocyst was defined as having a grade of at least 3BB, including 3/4/5AA, AB, BA, or BB.^22^ Fresh embryo transfer was performed on either day 2 (cleavage‐stage embryo) or day 5 (blastocyst stage). One or two embryos were transferred in each cycle. Blastocysts remaining after embryo transfer were stored at a low temperature using the vitrification technique and used for frozen embryo transfer (FET) in the future cycle. The protocols for FET included the natural cycle and the hormone replacement therapy cycle with transdermal estradiol and vaginal progesterone. The urine hCG test was carried out in 9 days after embryo transfer.

### 2.4 Outcomes

The numbers of punctured follicles, retrieved oocytes, fertilized oocytes, cleavage embryo, and blastocysts and its quality were evaluated in each of three groups (small, medium, and large follicles). Oocyte retrieval rate was expressed as a ratio of the number of retrieved oocytes to the number of punctured follicles. Fertilization rate was expressed as the ratio of the number of fertilized oocytes to the number of retrieved oocytes under cIVF. Under ICSI, fertilization rate was calculated as the number of fertilized oocytes to the number of MII oocytes because only MII oocytes underwent ICSI. Cleavage rate and blastocyst formation rate were expressed as a ratio of the number of cleavage embryos or blastocysts to the number of fertilized oocytes. Clinical pregnancy was defined as the detection of a gestational sac by ultrasonography. Pregnancy rate was expressed as the ratio of the number of pregnant cycles to the number of embryo transfer cycles. If two embryos derived from the different follicular size groups were transferred, such cases were excluded from the analysis of pregnancy rate.

## 3 RESULTS

A total of 176 patients (314 cycles) underwent cIVF, ICSI or split, respectively (Table 1). The prevalence of asthenozoospermia and oligospermia was significantly higher in the ICSI group than in the cIVF group. Consequently, the sperm concentration and sperm motility were significantly lower in the ICSI group than in the cIVF group. There were no significant differences of other backgrounds between three groups. In total, 3377 follicles were individually punctured, and 1482 oocytes were retrieved (Table 2). As shown in Table 2, the oocyte retrieval rates from small and medium follicles were significantly lower than the retrieval rate from large follicles. Furthermore, the retrieval rate was lower for small follicles than for medium follicles.

**TABLE 1:** Number of patients, cycles, and their backgrounds who underwent cIVF, ICSI, and split

	cIVF	ICSI	Split	Total
No. of patients	104	56	19	179
No. of cycles	175	111	28	314
Patient age (mean ± SD)	38.0 ± 4.6	38.1 ± 4.7	36.4 ± 3.7	37.9 ± 4.6
Patient's complications				
Myoma/adenomyosis (%)	15.4	14.3	15.8	15.1
Endometriosis (%)	10.6	8.9	26.3	11.7
PCOS (%)	2.9	10.7	5.3	5.6
Tubal factor (%)	17.3	5.4	5.3	12.3
Unilateral obstruction (%)	15.4	5.4	5.3	11.2
Bilateral obstruction (%)	1.9	0.0	0.0	1.1
Male factor (%)	12.5	33.9^a^	15.8	19.6
Asthenozoospermia (%)	3.8	17.9^b^	10.5	8.9
Oligospermia (%)	9.6	26.8^b^	5.3	10.6
Sperm findings				
Sperm concentration (10^6^/mL) (mean ± SD)	7082.8 ± 4475.4	4730.9 ± 3778.3^a^, ^c^	6758.6 ± 4008.8	6226.2 ± 4331.2
Sperm motility (%) (mean ± SD)	60.3 ± 14.6	50.3 ± 17.8^a^	58.9 ± 14.1	56 ± 16.4

^a^ *P* < .01 vs cIVF.

^b^ *P* < .05 vs cIVF.

^c^ *P* < .05 vs split.

**TABLE 2:** Oocyte retrieval rate according to follicular size

	Small follicles	Medium follicles	Large follicles	Total
No. of follicles punctured	1233	920	1224	3377
No. of oocytes retrieved	318	472	692	1482
Oocyte retrieval rate (%)	25.8^a^, ^b^	51.3^a^	56.5	43.9

^a^ *P* < .05 vs large follicles.

^b^ *P* < .05 vs medium follicles.

Table 3 shows the outcomes of 870 oocytes that underwent cIVF. The fertilization rates were significantly lower for oocytes from small and medium follicles than for oocytes from large follicles. Follicular size did not have a significant effect on the cleavage rate, the formation rate of blastocyst or blastocyst quality. To calculate the pregnancy rate, we analyzed the transfer of one or two embryos derived from the same follicular size group. In total, 144 cycles (162 embryos) were included in the analysis. The pregnancy rate and spontaneous abortion rate were not significantly affected by follicular size.

**TABLE 3:** Outcomes of oocytes that underwent cIVF

	Small follicles	Medium follicles	Large follicles	Total
No. of oocyte	190	274	406	870
No. of oocyte fertilized	102	156	269	527
Fertilization rate (%)	102/190 (53.7)^a^	156/274 (56.9)^a^	269/406 (66.3)	527/870 (60.6)
Cleavage rate (%)	100/102 (98.0)	151/156 (96.8)	265/269 (98.5)	516/527 (98.5)
Formation rate of blastocyst (%)	28/102 (27.5)	40/156 (25.6)	96/269 (35.7)	164/527 (31.1)
Formation rate of good quality blastocyst (%)	16/102 (15.7)	26/156 (16.7)	68/269 (25.3)	110/527 (20.9)
No. of embryo transfer cycle	28	32	84	144
No. of embryo transferred	30	37	95	162
Clinical pregnancy rate / cycle (%)	5/28 (17.9)	6/32 (18.8)	10/84 (11.9)	21/144 (14.6)
Spontaneous abortion rate (%)	0/ 5 (0)	1/6 (16.7)	3/10 (30.0)	4/21 (19.0)

^a^ *P* < .05 vs large follicles.

Table 4 shows the outcomes of 612 oocytes that were allocated to receive ICSI. The proportion of the retrieved oocytes that were mature (MII) was significantly lower for oocytes from small follicles than for oocytes from large follicles. Because only MII oocytes were used for sperm injection in ICSI cycle, the fertilization rate was calculated as the ratio of fertilized oocytes to the number of MII oocytes. The fertilization rate was not significantly affected by follicular size. As was observed in cIVF‐oocytes, follicular size did not have a significant effect on the cleavage rate, the formation rate of blastocyst, blastocyst quality, pregnancy rate, or spontaneous abortion rate.

**TABLE 4:** Outcomes of oocytes that underwent ICSI

	Small follicles	Medium follicles	Large follicles	Total
No. of oocyte	128	198	286	612
MII oocyte (%)	80/128 (62.5)^a^	149/198 (75.3)	230/286 (80.4)	459/612 (75.0)
No. of oocyte fertilized	64	115	178	357
Fertilization rate (%)	64 / 80 (80.0)	115 / 149 (77.2)	178 / 230 (77.4)	357/459 (77.8)
Cleavage rate (%)	62 / 64 (96.9)	111/115 (96.5)	177/178 (99.4)	350/357 (98.0)
Formation rate of blastocyst (%)	27/64 (42.2)	45/115 (39.1)	60/178 (33.7)	132/357 (37.0)
Formation rate of good quality blastocyst (%)	17/64 (26.6)	27/115 (23.5)	38/178 (21.3)	82/357 (23.0)
No. of embryo transfer cycle	22	33	55	110
No. of embryo transferred	24	36	74	134
Clinical pregnancy rate / cycle (%)	2/22 (9.1)	3/33 (9.1)	9/55 (13.8)	14/110 (12.7)
Spontaneous abortion rate (%)	1/ 2 (50.0)	1/3 (33.3)	1/9 (11.1)	3/14 (21.4)

^a^ *P* < .05 vs large follicles.

## 4 DISCUSSION

In this study, by culturing oocytes individually and monitoring the developmental outcomes up to the blastocyst stage, we found that the developmental capacity of fertilized oocytes under both cIVF and ICSI is not related to follicular size in Japanese women. Although small follicles contain fewer mature oocytes than large follicles, they can grow into blastocysts as well as oocyte from large follicles provided that they are fertilized. Therefore, our study shows the usefulness of puncturing not only large follicles, but also small follicles under COH cycle.

We found a significantly lower retrieval rate for oocytes from small follicles than for oocytes from large follicles, which is consistent with previous reports.^5^, ^6^, ^14^ This may be due to the difference in the attachment of the cumulus oocyte complex (COC) to the follicular wall between small and large follicles. The COC is embedded in the follicular wall in small follicles. As the follicle develops, the COC protrudes toward the antral cavity.^23^ Therefore, COCs in large follicles were more easily detached from the follicular wall by aspiration than were those in smaller follicles.

Under cIVF, the fertilization rare in oocytes from small follicles was significantly lower than that from large follicles, which is consistent with previous reports.^11^, ^24^ However, under ICSI, the fertilization rate was not affected by follicular size as reported previously.^11^, ^14^ Because oocytes undergoing cIVF were not denuded, their maturity was unclear. On the other hand, oocytes undergoing ICSI needed to be denuded for sperm injection, which made it possible to observe their maturity. In the ICSI cycle, the proportion of mature oocytes was lower for oocytes from small follicles than for oocytes from large follicles. There is no doubt that these proportions were not markedly different in the retrieved oocytes under cIVF cycle. Therefore, the decreased fertilization rate of oocytes from small follicle under cIVF may be due to a lower proportion of mature oocytes. This is supported by previous findings that the proportion of mature oocytes was correlated with follicular size.^5^, ^10^, ^14^ In other words, oocytes from small follicles have same potential of fertilization as oocytes from large follicles if they are matured.

Our study also examined whether the developmental capacity of the fertilized oocytes is correlated with the follicular size. Some studies have found a correlation between oocyte developmental capacity and follicular size,^7^, ^9^ while others did not.^5^, ^10^, ^11^, ^24^ Therefore, their relationship is still controversial. However, these reports assessed it according to the cleavage rate or the quality of cleavage‐stage embryos, but not blastocysts. Recently, transfer of human embryo at the blastocyst stage is becoming more common in the practice of assisted reproduction technology because blastocyst it is associated with a higher implantation rate than transfer of cleavage‐stage embryos.^12^ In addition, it should be noted that only 30% of cleavage‐stage embryos can reach the blastocyst stage.^25^ Therefore, the developmental capacity of oocyte should be determined by monitoring the developmental process up to the blastocyst stage. Then, we need to find out whether it correlates with follicular size. In our study, there was no correlation between follicular size and the formation rate or blastocyst quality, showing that oocytes from small follicles have the comparable capacity of blastocyst formation with good quality as those from large follicles. These our findings are similar to those of Wirleitner et al.^14^ However, their study was limited to oocytes fertilized under ICSI, and thus lacked data on oocytes that underwent cIVF. In addition, they did not examine blastocyst quality. Therefore, our study is the first showing that there is no correlation between follicular size and development capacity of oocytes under both cIVF and ICSI. Taken together, these results indicate that even oocytes from small follicles can grow into blastocysts as well as oocytes from large follicles if they can be fertilized. Furthermore, not only blastocyst formation rate, but pregnancy rate was not different by follicular size, which is consistent with previous report.^14^ In COH cycle, several blastocysts are often obtained in one cycle. Therefore, selection of the best embryo to be transferred is important. Out data suggest that the follicular size that blastocyst was derived from cannot be a criterion for selecting the embryo to be transferred.

It should be noted that the efficiency of ART is different between races.^15^, ^16^ To our best knowledge, this is the first report examining the relationship between the developmental capacity of oocytes and the follicular size under COH in Japanese women although Japan is the largest user of ART worldwide in terms of annual number of treatment cycles performed.^1^, ^17^, ^18^ Recently, Teramoto et al^26^ examined the size/development capacity relationship in Japanese women although their study was conducted under the natural non‐stimulated ART cycle, not under the COH cycle. They showed that the proportion of MII oocytes was only 25.4% in small follicles, which is much lower than that observed in our study (62.5%, Table 4). This might be due to the difference of follicular stage between natural and COH cycle. In a natural cycle, only one mature follicle appears to ovulate while others go to atresia.^3^ On the other hand, in COH, follicles are stimulated by high levels of FSH, which suppresses follicular atresia and induces the growth of multiple follicles of different sizes. Therefore, even if the follicles under COH are as small as the non‐dominant follicles under non‐stimulated cycle, they include growing follicles.^3^ This might be the reason that mature oocytes could be more obtained from small follicles under COH cycles than under non‐stimulated cycle. Furthermore, it is interesting to note that the blastocyst formation rate of fertilized oocytes from small follicles under non‐stimulated cycle was lower than that from large follicles.^26^ This is not consistent with our results. They used only MII oocytes for insemination and analyzed the blastocyst formation rate by calculating the number of blastocysts relative to the fertilized oocytes as we did. Therefore, even if oocytes from small follicles show MII status and could be fertilized, their developmental capacity into blastocyst is different between non‐stimulated cycles and COH cycles.

This study revealed the relationship between the developmental capacity of oocytes up to blastocyst stage and follicular size under both cIVF and ICSI in Japanese women. Although the fertilization rate by cIVF is low in oocytes from small follicles due to a lower proportion of mature oocytes, their development potential is comparable to that of oocytes from larger follicles if they could be fertilized by either cIVF or ICSI. Therefore, it is worth to puncture small follicles which are grown under COH. Because Japan has the largest number of ART cases in the world, out study should provide useful information to many clinicians and patients.

## DISCLOSURE

Conflict of interest: All authors have no conflict of interest.

Ethics Statement: Informed consent was obtained from all the patients in this study. The study design was reviewed and approved by the institutional review board of Yamaguchi University Hospital.
